# Design and Development of a Linked Open Data-Based Health Information Representation and Visualization System: Potentials and Preliminary Evaluation

**DOI:** 10.2196/medinform.3531

**Published:** 2014-10-25

**Authors:** Binyam Tilahun, Tomi Kauppinen, Carsten Keßler, Fleur Fritz

**Affiliations:** ^1^Institute for Medical InformaticsUniversity of MünsterMünsterGermany; ^2^Cognitive SystemsUniversity of BremenBremenGermany; ^3^Center for Advanced Research on Spatial Information and Department of GeographyHunter College, City University of New YorkNew York, NYUnited States

**Keywords:** Linked Open Data, Semantic Web, ontology, health information systems, HIV, WHO, public health, public health informatics, visualization

## Abstract

**Background:**

Healthcare organizations around the world are challenged by pressures to reduce cost, improve coordination and outcome, and provide more with less. This requires effective planning and evidence-based practice by generating important information from available data. Thus, flexible and user-friendly ways to represent, query, and visualize health data becomes increasingly important. International organizations such as the World Health Organization (WHO) regularly publish vital data on priority health topics that can be utilized for public health policy and health service development. However, the data in most portals is displayed in either Excel or PDF formats, which makes information discovery and reuse difficult. Linked Open Data (LOD)—a new Semantic Web set of best practice of standards to publish and link heterogeneous data—can be applied to the representation and management of public level health data to alleviate such challenges. However, the technologies behind building LOD systems and their effectiveness for health data are yet to be assessed.

**Objective:**

The objective of this study is to evaluate whether Linked Data technologies are potential options for health information representation, visualization, and retrieval systems development and to identify the available tools and methodologies to build Linked Data-based health information systems.

**Methods:**

We used the Resource Description Framework (RDF) for data representation, Fuseki triple store for data storage, and Sgvizler for information visualization. Additionally, we integrated SPARQL query interface for interacting with the data. We primarily use the WHO health observatory dataset to test the system. All the data were represented using RDF and interlinked with other related datasets on the Web of Data using Silk—a link discovery framework for Web of Data. A preliminary usability assessment was conducted following the System Usability Scale (SUS) method.

**Results:**

We developed an LOD-based health information representation, querying, and visualization system by using Linked Data tools. We imported more than 20,000 HIV-related data elements on mortality, prevalence, incidence, and related variables, which are freely available from the WHO global health observatory database. Additionally, we automatically linked 5312 data elements from DBpedia, Bio2RDF, and LinkedCT using the Silk framework. The system users can retrieve and visualize health information according to their interests. For users who are not familiar with SPARQL queries, we integrated a Linked Data search engine interface to search and browse the data. We used the system to represent and store the data, facilitating flexible queries and different kinds of visualizations. The preliminary user evaluation score by public health data managers and users was 82 on the SUS usability measurement scale. The need to write queries in the interface was the main reported difficulty of LOD-based systems to the end user.

**Conclusions:**

The system introduced in this article shows that current LOD technologies are a promising alternative to represent heterogeneous health data in a flexible and reusable manner so that they can serve intelligent queries, and ultimately support decision-making. However, the development of advanced text-based search engines is necessary to increase its usability especially for nontechnical users. Further research with large datasets is recommended in the future to unfold the potential of Linked Data and Semantic Web for future health information systems development.

## Introduction

Information is a foundation for effective decision-making. This information need is even more critical in public health organizations to support areas such as epidemiologic surveillance, health outcome assessment, program evaluation and performance measurement, public health planning, and policy analysis [1]. In order to satisfy this, we need better and more flexible health data representation, analysis, querying, and visualization methods. The amount of available online health data both in structured and unstructured formats is constantly increasing. The World Health Organization (WHO), for example, has established a data repository providing access to over 50 datasets on priority health topics including mortality and prevalence of human immunodeficiency virus infection/acquired immunodeficiency syndrome (HIV/AIDS) in different WHO regions [2]. Moreover, the United Nations [3] and the Centers for Disease Control and Prevention (CDC) [4] have online data repositories on the different indicators for different countries.

While these are important initiatives to publish health data online, there has been relatively little attention paid to data representation methods in most health data portals so far [5]. Current data representation and distribution methods with only tabular formats, such as comma-separated values (CSV), PDF, and Excel—and little metadata—makes health information integration, comparison, and reuse very difficult. Additionally, even though different indicators have relationships to each other, the datasets are not linked in most portals. Vocabularies and data formats are inconsistent, which makes finding, assembling, and normalizing these datasets time consuming and prone to errors [6].

Exploiting the different kinds of public health information about a given topic is a challenging task because data is spread across different platforms in heterogeneous formats. Better data management methods and tools are required to move from a Web of documents, only understandable by human users, to a Web of Data in which information is expressed in a format that can be read and used by machines. This would enable us to find, share, and integrate information more easily [7].

Linked Data, as explained by Tim Berners-Lee [7], is a method to publish structured data by using standard Web technologies to connect related data and make them accessible on the Web. The Linked Data publishing pattern uses HTTP uniform resource identifiers (URIs) for identifying data items, the Resource Description Framework (RDF) for describing data, and links to describe the relationships. Other standards used in Linked Open Data (LOD) applications include Resource Description Framework Schema (RDFS) for describing RDF vocabularies, and SPARQL Protocol and RDF Query Language (SPARQL) for querying RDF graphs [8].

The primary goal of the Linked Data initiative is to make the World Wide Web (WWW) not only useful for interlinking documents, but also for sharing and interlinking data [9]. The movement is driven by the hypothesis that these technologies could revolutionize global data sharing, integration, and analysis, just like the classic Web-revolutionized information sharing and communication over the last two decades. However, to our knowledge there are not many studies on the potential of LOD for public health data management.

Motivated by the universal hypothesis of Linked Data to revolutionize data sharing, integration, and analysis, the main objectives of this work are (1) to test the potential of LOD for health data representation and visualization, (2) to identify the available technologies and tools for Linked Data-based health information system development, and (3) to evaluate the usability level of LOD-based systems by end users.

In this paper, we present the development of the system from data modeling to visualization and potential LOD tools available for development. Identifying the tools and testing the potential of LOD will be helpful as an input to the health informatics and Semantic Web community in the research effort to find ways to represent data in a flexible manner.

## Methods

### Overview

Our methodology was “Integration-oriented development and evaluation” in the sense that we used the available LOD tools to develop the system and then we reflected on the development process, the potentials, and finally on usability for end users. We gave special emphasis to the data management process as efficient data management and conversion is the backbone of the LOD-based system development [10]. We used the RDF for data representation, Fuseki triple store for data storage, and Sgvizler for information visualization. Additionally, we integrated a SPARQL query interface for interacting with the data. We primarily used the WHO health observatory dataset to test the system. All the data were represented using RDF and interlinked with other related datasets on the Web of Data using Silk [11], a link discovery framework for Web of Data. A preliminary usability assessment was conducted following the System Usability Scale (SUS) method. The final revised SUS questionnaire used for the evaluation is shown in Multimedia Appendix 1. The details, with more focus on the data management process, are explained throughout this paper.

### Data Sources

The dataset for this work was retrieved from the WHO global health observatory data repository [2]. The data used covered the years from 1990 to 2010. Missing data for some years were complemented with data from other similar official sources, such as the United Nations program for HIV/AIDS (UNAIDS) [3] and country-specific official sources like the national AIDS resource centers of each African country. From those databases, HIV statistical data, as well as additional location and total population information, were extracted for sub-Saharan African countries. Most of the data were in Microsoft Excel and CSV formats. All the data were converted and prepared in Excel using the Excel2RDF [12] converter. For the enrichment, DBpedia, Bio2RDF and LinkedCT were used as sources. For data license, all our published Linked Data adheres to the original data publisher’s license and terms of use.

### Data Modeling and Conversion

Shared vocabularies are a key to enable interoperability in healthcare systems by providing an agreed-upon terminology that can be looked up through URIs that cannot be referenced [13]. We have identified potential health, statistical, spatial, and time vocabularies and ontologies to share the data in a reusable way and then mapped them to the external ontologies using predicates (see Table 1). We used the common RDF [14], RDFS [15], Web Ontology Language (OWL) [16], friend of a friend (FOAF) [17], and Data Cube [18] vocabularies for data annotation. Those are standard vocabularies to represent data in LOD by expressing relationships between the data. We use the Data Cube vocabulary for all the statistical data to represent, not only the numbers, but also advanced metadata with space and time dimensions of the observation. Some of the standard predicates were replaced with more generic elements from the Data Cube vocabulary (eg, qb:prevalence instead of qb:observation) to make them more understandable to health professionals and healthcare managers. We assume that using some of the terms that are already known by health professionals will make the system more usable and easily adaptable. After identifying the ontologies and vocabularies, the original data was converted in a semi-automated way to avoid information loss. Conversion using Excel2RDF is done by selecting the range of data values and headers from the spreadsheet that are to be converted. Then, the headers are fed into the mapping wizard, which assists the mappings of row/column concepts to RDF vocabularies. Excel data triplication using Excel2RDF is discussed by Pesce et al [19].

**Table 1:** Different domain ontological vocabularies and predicates reused for modeling data in the conversion process.

Domain	Ontological vocabulary	Predicate
**Health**		
	prefix MeSH	Interlinked with <owl:sameAs>
	prefix Diseasome	Interlinked with <owl:sameAs>
	prefix dbpedia	Interlinkedwith<owl:sameAs>
**Spatial**		
	Prefix geo	geo:lat, geo:long
	prefix dcterm	dcterm:country
**Statistical**		
	prefix qb	qb:prevalence qb:slice qb:item
	prefix scovo	qb:prevalence qb:slice qb:item
**Time series**	prefix time	Time: year ( from 1990-2010)

###  Data Storage

The main difference between existing health information system development and Linked Data-based systems is the way data is represented and stored. Current systems mostly use tabular formats (eg, Excel, CSV) or relational database systems such as Oracle. Linked Data-based systems, however, usually build on triple stores as their main data storage. This triple-based representation enables integration of data available from various sources without the need for physical storage of the RDF triple that corresponds to the relational data [20]. These systems provide data management and data access via application programming interfaces (APIs) and query languages to RDF data. For this work, we used the Fuseki triple store [21]. It provides representational state transfer (REST)-style SPARQL HTTP Update, SPARQL Query, and SPARQL Update using the SPARQL protocol over HTTP [22].

###  Data Enrichment

The primary intention of representing health data using the LOD approach is to be able to discover and link health data from different sources and use them in new applications. Interlinking data from our RDF datasets to other datasets, which are already in the LOD cloud, was challenging. It requires identification of similar link types in our datasets, and then finding suitable matching links in external datasets. Zevari et al point out similar challenges in link discovery in health datasets [23].

**Figure 1:**
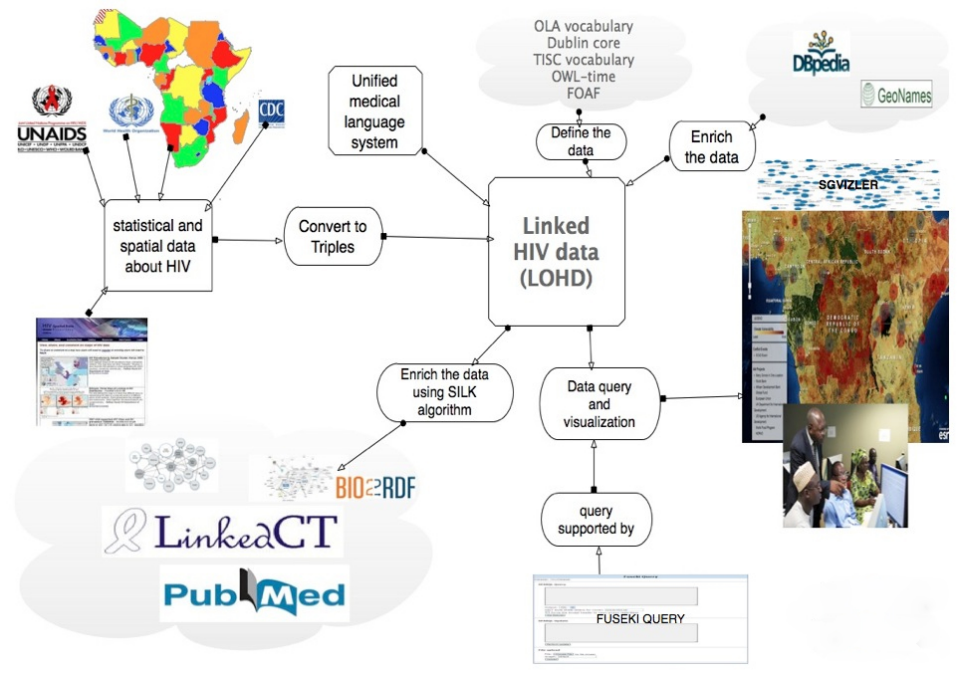
The overall workflow diagram for the methodology from the data conversion, data interlinking, and data query to visualization.

In our data enrichment, we used both manual and automatic methods. We manually enriched the dataset with links to some sources such as DBpedia, while large numbers of links to sources such as Bio2RDF were generated automatically. The enrichment is based on owl:sameAs relations, which interconnect different identifiers for the same real-world item across different datasets (eg, DBpedia:Ethiopia owl:sameAs geonames:7733022). Such a sameAs-link references different identifiers for the same real-world entity—Ethiopia, in our example—from different sources [10]. We enriched the data with links to data sources generated by related initiatives such as Bio2RDF [24], LinkedCT [25], Pubmed [26], and other geospatial and health-related initiatives using standard RDF and Unified Medical Language System (UMLS) vocabularies. We used the Silk Link Discovery Framework [27] for automatic link discovery and to provide the built-in Fuseki query interface to access the data. To access the target data, we first configured access parameters to the target dataset endpoints using the <DataSource> directive. The only mandatory data source parameter is the endpoint URI. By specifying the source and destination endpoints on target datasets, we interlinked the data. In total, we retrieved 5312 data elements to be added to the system. Additionally, we implemented a visualization interface over the triple store using Sgvizler [28], a JavaScript library which renders the results of SPARQL queries as charts or HTML elements [29]. Figure 1 gives an overview of the overall methodology.

## Results

### Overview

We developed a Linked Open Health Data (LOHD) system that integrates spatial and statistical health data from various sources. In the system, users can query HIV-related information about African countries and the system will support them in querying and visualizing the data in both space and time.

###  LOHD System Architecture

**Figure 2:**
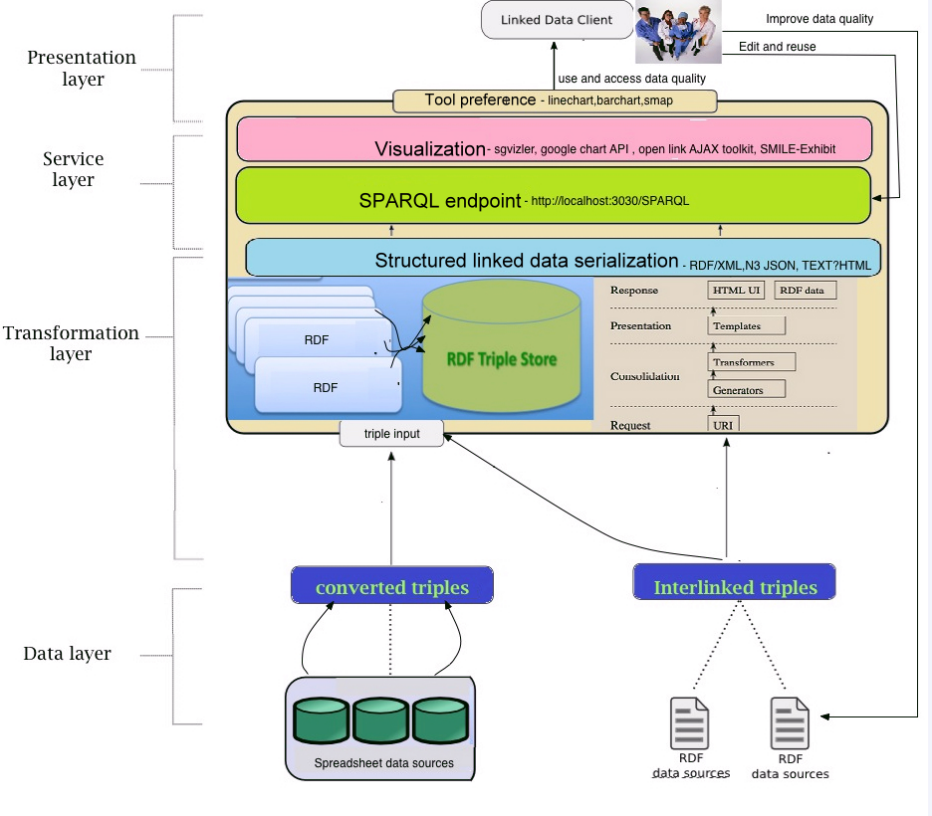
LOHD System architecture with all the four layers from the data representation layer to presentation layer and the corresponding LOD tools.

For the system development, we preferred a multilayer architecture, which provides flexibility and reusability. For example, data management, query processing, and visualization are logically separate processes. The advantages of a multilayer architecture have been discussed in the literature in detail [27-29]. By breaking up the system into a hierarchy, different layers can be developed sequentially and modified asynchronously without affecting the entire system architecture [10]. The architecture of our system is composed of 4 main layers (see Figure 2): (1) the data layer, (2) the transformation layer, (3) the service layer, and (4) the presentation layer. The data layer stores the converted and interlinked data. The transformation layer is the processing layer where every SPARQL query is processed using crawling pattern to localize data from the Web of Linked Data. The service layer controls the data access and bridges the client to the server via service protocols. The presentation layer allows the users to interact with the services using either retrieval or visualization tools. All the system architecture layers and the underlying LOD application tools are shown in Figure 2.

###  Visualization

Coherent LOD visualizations enable nontechnical users to use the Web of Data [30] and increase the usability and accessibility of Linked Data-based systems [31]. In most Linked Data-based systems, the user is expected to write SPARQL queries, which is challenging for nontechnical users. To overcome those challenges, we integrate a live visualization interface using Sgvizler. Once the query is selected, the users have the option to choose the visualization method for the data output. All the visualization methods available on Sgvizler are supported by our system. In the following sample queries, we show some of the visualizations based on spatial or temporal queries.

### Time Series Visualization of Linked Data

**Figure 3:**
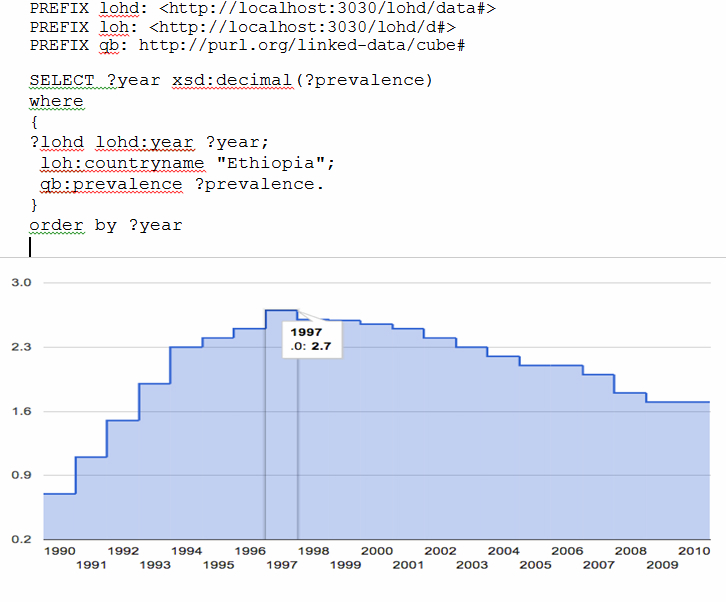
Time series visualization of HIV prevalence in Ethiopia from the years 1990-2010. To visualize other countries, substitute the country name in the query.

Time series visualizations help to display patterns and trends that are not readily apparent in the numbers themselves. In traditional databases, time series visualization is mostly done by external applications which are cumbersome and time consuming. But in Linked Data-based systems, you can write your query and choose the visualization type from the drop-down menu. Figure 3 shows the trend of HIV prevalence in Ethiopia, as an example, and the system automatically shows the live visualization of the trend for the requested year.

### Geographical Visualization of Linked Data

**Figure 4:**
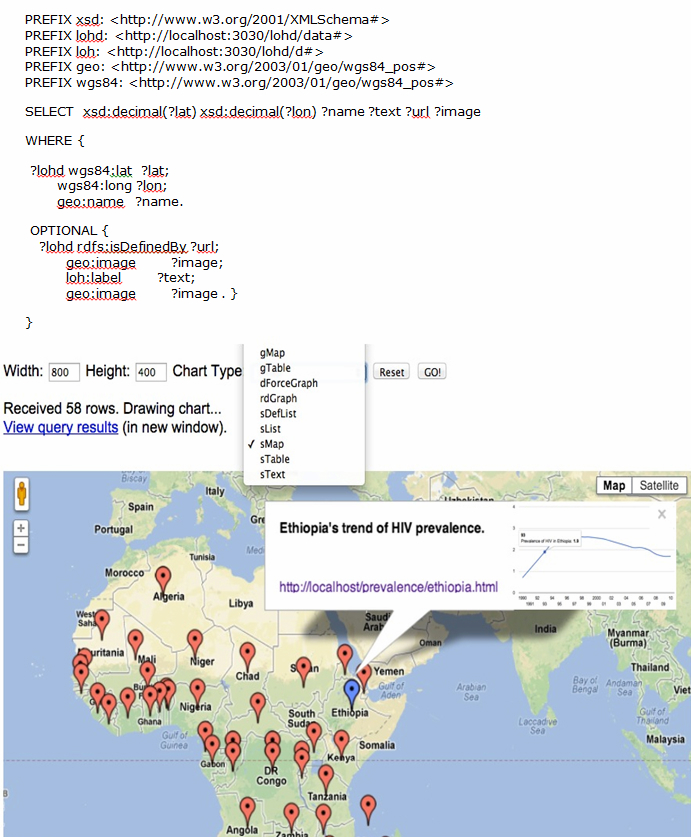
Location-based visualization of HIV prevalence in sub-Saharan Africa. The health-related data and the time series graph are displayed by clicking on the map of the country.

Location is becoming a basic attribute for health data [32]. Location-based visualizations are mostly difficult using traditional databases unless they are exported to geographic information system (GIS) software for further analysis. In LOD-based systems, location-based visualizations are facilitated by the ability to write queries and choose the visualization method. Figure 4 shows an example where the visualization shows the prevalence of HIV based on each country’s location on the African map. When someone clicks on the icon of the country, it will show the basic information about the country and the trend of HIV for the specified time period in the query.

### Indicator-Based Visualization of Linked Data

**Figure 5:**
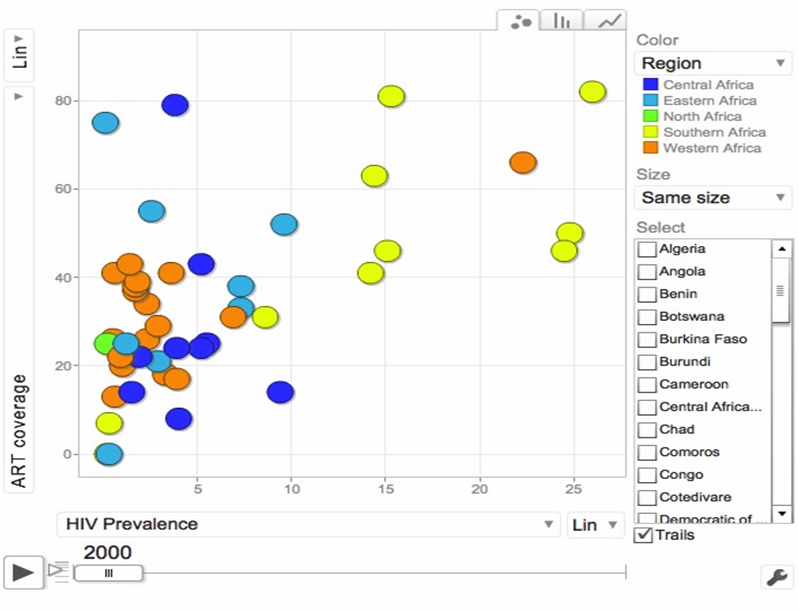
Indicator-based correlation visualization over LOHD system of HIV prevalence and ART coverage versus time.

Indicators are the basic components of any health data. Most international disease prevalence comparisons and local-level reporting are done using indicators in a specific period of time. LOD-based systems support queries with different indicators—such as HIV prevalence rate by country or region, antiretroviral therapy (ART) coverage rate, population or gross domestic product (GDP)—and make a correlation analysis between those variables over time. In Figure 5, we show a 3-dimensional correlation analysis with time series animation.

### Evaluation

The system was evaluated in a small-scale user study to get feedback from healthcare data managers and users regarding the usability and learnability of the system. A total of 19 participants were selected for this evaluation, both with a technical and nontechnical background. The participants had no relationship with the investigator and the selection was done purposefully to ensure we recruited participants who currently work on health data management, and to get a proportional mix of different professions. Of the 19 selected participants, 17 of them responded to the questionnaire (89%). The technical participants (9/17, 53%) were data managers with IT backgrounds, health information system developers, and system administrators in different healthcare organizations in Africa. The nontechnical participants (8/17, 47%) were public data users, such as demographic data managers, doctors, and public health professionals. The evaluation was done based on the SUS with some wording amended, tailored for our participants (see Multimedia Appendix 1). In the evaluation, we were interested in the feedback from the participants on the query-based data access. The Linked Data search engine was not provided to participants, given its early stage of development for complex query request. The SUS is mostly regarded as a quick and easy way to conduct a usability assessment [33]. Even though the tool is self-described as “quick and dirty”, it has been evaluated in many studies (more than 600 articles) as valid and reliable [34]. Based on the SUS scoring criteria, the final calculated score was 82, which is well above the average SUS score of 68. Table 2 summarizes the evaluation responses for each criteria of the system usability.

Additionally, 2 open-ended questions were asked to the users to better understand their views and their specific requirements for using the system. The frequent answers for those questions can be explained by dividing them into 2 groups. The participants with technical backgrounds were relatively happy and 8 out of 9 (89%) of them mentioned that such systems would be useful in the future. The nontechnical participants (8/8), however, mentioned that the system was not easy to use. This is understandable seeing that the current Linked Data tools demand writing queries. Publishing the data in machine-understandable form and making live visualization without having to use external applications were the most frequently mentioned benefits of the system by the participants of the evaluation (15/17, 88%). The need to write queries in the optional interface and identifying the appropriate visualization tool were reported as being the difficult aspects of such systems by 16 of the 17 (94%) participants.

**Table 2:** *.* SUS evaluation criteria and participant response (n=17).

SUS evaluation criteria	Strongly agree, n (%)	Agree, n (%)	Neutral, n (%)	Disagree, n (%)	Strongly disagree, n (%)
I think that I would like to access my data this way.	10 (59)	3 (18)	-	4 (24)	-
I found the system unnecessarily complex.	2 (12)	5 (29)	-	10 (59)	-
I thought the system was easy to use.	4 (24)	5 (29)	-	7 (41)	1 (6)
I think that I would need the support of a technical person to be able to use this system.	6 (35)	1 (6)	-	7 (41)	3 (18)
I found the various functions in this system well integrated.	12 (71)	2 (12)	-	3 (18)	-
I thought there was too much inconsistency in this system.	3 (18)	2 (12)	-	10 (59)	1 (6)
I would imagine that most people would learn to use this system very quickly.	6 (35)	1 (6)	2 (12)	6 (35)	3 (18)
I found the system very cumbersome to use.	4 (24)	2 (12)	-	11 (65)	1 (6)
I felt very confident using the system.	12 (71)	-	-	5 (29)	-
I needed to learn a lot of things before I could get going with this system.	7 (41)	1 (6)	-	9 (53)	-

## Discussion

### Principal Findings

We developed a Linked Data-based health information representation, querying, and visualization system. We used the system to represent and store the data, facilitating flexible queries and different kinds of visualizations. There are other ongoing efforts to convert healthcare- and life science-related datasets to a Linked Data cloud such as Linked Open Drug Data (LODD), LinkedCT, Open Biomedical Ontologies (OBO), and the World Wide Web Consortium’s (W3C) Health Care and Life Sciences working groups [31,32]. Thanks to such initiatives and recently developed Semantic Web tools, converting data to RDF has become straightforward. However, just converting the data to RDF and publishing it online is not enough [35,36]. The main difficulty is to integrate the data representation methods to application-level tools and make them usable for health information consumers in a shared, semantically meaningful, easily discoverable, and reusable manner.

In our system, we represented the health data with its important dimensions—magnitude, time, and space—in the form of RDF and we used both manual and automatic interconnection methods to enrich the data. We integrated visualization and retrieval methods for the data to make data visualization and retrieval possible with already available tools. There was a similar initiative by Zappa et al to integrate mutation data in the LOD cloud [35]. The methodology we follow for development is similar except that they use another tool for the data conversion. What makes our work different is that in addition to converting the data and making it available in RDF, we focus on integrating additional query and visualization interface tools to make the system more usable, especially for nontechnical users.

Our system development method was integration oriented in the sense that it reflects the way to convert the different dimensions of the data to Linked Data and integrate them with already developed tools, enabling the system to support information access. In selecting our tools, we found out that RDF is currently a robust data model to represent data with metadata [14,37] that gives the opportunity of integrating data and availing data for query. Our selection of Sgvizler for visualization was motivated by its current support of different types of visualization and its integration with HTML webpages by letting the user specify queries of interest [29]. One of the difficulties we noticed here is that for complex queries, Sgvizler is relatively slow. This may make it difficult to use for big data and complex query-based systems. Nonetheless, we believe that advanced-level, live correlation visualization of certain disease trends in space and time dimensions from different sources is one of the biggest promises of Linked Data-based systems in the future.

Measuring the degree of advancement that a Linked Data representation brings to public health data is difficult to quantify. Nonetheless, from the technology perspective, the data becomes search engine discoverable and machine understandable, which addresses the main issues of the current health data silos problem [38]. While Linked Data and Semantic Web technologies are not as mature as other database technologies, they present a promising alternative in public health information portal development. A good example that can explain this is the data representation scheme in the World Bank database [39], which includes both a portal for downloading data as Excel or PDF files, as well as a Linked Data version for downloading the data as RDF with the ability to query their endpoints. The main advantage of having Linked Data as an additional option in the World Bank database can be seen in the results of search engine results. If you input “Prevalence of HIV in Egypt” and “GDP of Egypt” into search engines, we can clearly see the data representation limitation of health portals. Since the World Bank data is represented in a machine-understandable and search engine-discoverable way, you can see the graphs and additional descriptions, which are very useful for an end user searching for them.

The user evaluation of our system confirms the existing usability limitations of Linked Data mentioned by different authors [21,32,35,36,40]. Linked Data is currently mostly used by the Semantic Web community and other users with a strong technical background. To make the Linked Data-based systems more usable by end users, we need to develop enhanced tools that can avoid the need to write queries.

In our evaluation, 41% (7/17) of the participants (strongly agree and agree together) reported that they need the support of a technical person to use this system, which is high when compared to other system evaluations [4,33]. Yet this is an expected result given the current technical nature of data access in LOD when using queries. The promising result from the evaluation is that 70% (12/17) of the participants are confident in using and understanding the visualizations of the system. This indicates that the LOD-based representation of public health data offers a new perspective in the future of health data portal development.

### Limitations

There are some limitations in this work. Primarily, the amount of data we used is small to generalize the robustness of the LOD tools. As already outlined in different studies [41-43] Semantic Web technologies work well with small datasets but might not be the best option with big datasets. Secondly, our user evaluation was based on a small set of participants and the SUS scale, which has its own limitations, making generalization of the usability assessment result difficult.

For future research we recommend integrating and testing an advanced-level search engine to ensure that LOD-based systems are more usable outside the Semantic Web community. Additionally, implementing and testing a similar system with a big dataset by describing the data more robustly with domain-specific, additional ontological vocabularies, interlinking with more ontologies, and including more visualization options for grouped data is recommended. Moreover, implementation of advanced-level correlation analysis visualization from different sources will make LOD technology more interesting and usable by healthcare professionals.

### Conclusions

The system introduced in this article shows that LOD has a promising potential in the representation of complex health-related data. This is mainly due to its reusable and interoperable manner that can serve intelligent queries, and ultimately support decision-making. However, the development of advanced LOD search engines is necessary to increase its usability.

## Multimedia Appendix 1

Final revised SUS questionnaire for the evaluation.

## Abbreviations

API: application programming interface
ART: antiretroviral therapy
CDC: Centers for Disease Control and Prevention
FOAF: friend of a friend
GDP: gross domestic product
GIS: geographic information system
LOD: Linked Open Data
OWL: Web Ontology Language
RDF: Resource Description Framework
RDFS: Resource Description Framework Schema
REST: representational state transfer
SPARQL: SPARQL Protocol and RDF Query Language
SUS: System Usability Scale
UMLS: Unified Medical Language System
URI: uniform resource identifier
W3C: World Wide Web Consortium
WHO: World Health Organization
