# Supplementary material for: Design and Development of a Linked Open Data-Based Health Information Representation and Visualization System: Potentials and Preliminary Evaluation
Source: JMIR Med Inform. 2014 Oct 25;2(2):e31. doi: 10.2196/medinform.3531 (PMC4288106; doi:10.2196/medinform.3531)
Supplement: Supplementary file 1 [file medinform_v2i2e31_app1.pdf]

Appendix I- Questionnaire based on SUS

Overall System Usability Evaluation

1. I think that I would like to use this system frequently.

    (1) Strongly disagree (2) Disagree (3) Neutral (4) Agree (5) Strongly Agree

2. I found the system unnecessarily complex.

    (1) Strongly disagree (2) Disagree (3) Neutral (4) Agree (5) Strongly Agree

3. I thought the system was easy to use.

    (1) Strongly disagree (2) Disagree (3) Neutral (4) Agree (5) Strongly Agree

4. I think that I would need the support of a technical person to be able to use this system.

    (1) Strongly disagree (2) Disagree (3) Neutral (4) Agree (5) Strongly Agree

5. I found the various functions in this system were well integrated.

    (1) Strongly disagree (2) Disagree (3) Neutral (4) Agree (5) Strongly Agree

6. I thought there was too much inconsistency in this system.

    (1) Strongly disagree (2) Disagree (3) Neutral (4) Agree (5) Strongly Agree

7. I would imagine that most people would learn to use this system very quickly

    (1) Strongly disagree (2) Disagree (3) Neutral (4) Agree (5) Strongly Agree

8. I found the system very cumbersome to use.

    (1) Strongly disagree (2) Disagree (3) Neutral (4) Agree (5) Strongly Agree

9. I felt very confident using the system.

    (1) Strongly disagree (2) Disagree (3) Neutral (4) Agree (5) Strongly Agree

10. I needed to learn a lot of things before I could get going with this system

    (1) Strongly disagree (2) Disagree (3) Neutral (4) Agree (5) Strongly Agree

11. What is the difficult part of this system compared to the one you are using?

    Currently?

    ____

    ____

12. Do you think such systems are important for your public health data and platform?

    need? ___ If yes, why?

    ____

    ____

    ____
